# Supplementary material for: Assessing the Validity and Reliability of a Single Lumbar-Mounted IMU System for Gait Analysis
Source: Sensors (Basel). 2025 Dec 17;25(24):7643. doi: 10.3390/s25247643 (PMC12737096; doi:10.3390/s25247643)
Supplement: Supplementary file 1 [file sensors-25-07643-s001.zip › sensors-3956116-supplementary.pdf]

Table S1. Intra-rater measurements for Baiobit (Rater A)

Variables	1st measurement	2nd measurement	ICC		SEM	MDC(90%)	Bland-Altman Analysis		
	Mean (SD)	Mean (SD)	ICC	95% CI			Mean difference (SD)	95% CI	LOA superior - inferior
bai_stance_phase	60.31(2.23)	60.38(3.13)	0.63	0.35 to 0.81	0.49	1.13	-0.06(2.37)	0.84 to -0.96	4.58 to -4.7
bai_swing_phase	37.31(9.45)	39.61(3.13)	0.63	0.36 to 0.81	1.28	2.99	0.07(2.36)	0.97 to -0.83	4.7 to -4.56
bai_single_support_phase	39.93(2)	39.64(3.19)	0.53	0.22 to 0.75	0.63	1.46	0.29(2.59)	1.28 to -0.69	5.38 to -4.79
bai_double_support_phase	20.38(4.09)	20.76(6.27)	0.61	0.32 to 0.79	0.78	1.81	-0.38(4.74)	1.42 to -2.18	8.91 to -9.67
bai_stride_length	1.53(0.22)	1.49(0.21)	0.69	0.44 to 0.84	0.03	0.06	0.04(0.17)	0.11 to -0.02	0.37 to -0.28
bai_step_length	0.77(0.11)	0.75(0.11)	0.68	0.42 to 0.83	0.03	0.06	0.02(0.09)	0.06 to -0.01	0.19 to -0.14
bai_velocity	1.31(0.2)	1.32(0.2)	0.90	0.81 to 0.95	0.02	0.05	0(0.09)	0.03 to -0.04	0.17 to -0.18
bai_cadence	117.39(13.91)	121.1(11.57)	0.80	0.61 to 0.9	0.58	1.35	-3.72(7.47)	-0.88 to -6.56	10.92 to -18.36

Table S2. Intra-rater measurements for Baiobit (Rater B)

Variables	1st measurement	2nd measurement	ICC		SEM	MDC(90%)	Bland-Altman Analysis		
	Mean (SD)	Mean (SD)	ICC	95% CI			Mean difference (SD)	95% CI	LOA superior - inferior
bai_stance_phase	60.68(1.73)	60.68(1.58)	0.74	0.51 to 0.87	0.17	0.39	0(1.22)	0.46 to -0.47	2.39 to -2.39
bai_swing_phase	43.49(22.92)	39.34(1.59)	0.74	0.52 to 0.87	1.98	4.62	-0.02(1.22)	0.45 to -0.48	2.37 to -2.4
bai_single_support_phase	39.86(1.81)	39.63(1.83)	0.73	0.5 to 0.86	0.07	0.17	0.22(1.35)	0.74 to -0.29	2.87 to -2.42
bai_double_support_phase	21.1(3.5)	21.63(2.92)	0.74	0.52 to 0.87	0.32	0.76	-0.53(2.3)	0.34 to -1.4	3.97 to -5.03
bai_stride_length	1.5(0.18)	1.53(0.23)	0.71	0.48 to 0.85	0.11	0.25	-0.04(0.16)	0.02 to -0.1	0.27 to -0.34
bai_step_length	0.75(0.09)	0.77(0.12)	0.69	0.45 to 0.84	0.08	0.18	-0.02(0.08)	0.01 to -0.05	0.14 to -0.18
bai_velocity	1.33(0.2)	1.34(0.2)	0.91	0.81 to 0.95	0.02	0.04	-0.01(0.09)	0.03 to -0.04	0.16 to -0.18
bai_cadence	120.96(9.23)	120.24(12.66)	0.85	0.72 to 0.93	0.59	1.39	0.72(6.03)	3.01 to -1.58	12.54 to -11.1

Table S3. Intra-rater measurements for BTS (Rater A)

Variables	1st measurement	2nd measurement	ICC		SEM	MDC(90%)	Bland-Altman Analysis		
	Mean (SD)	Mean (SD)	ICC	95% CI			Mean difference (SD)	95% CI	LOA superior - inferior
bts_stance_phase	62(1.97)	61.7(2.55)	0.59	0.3 to 0.78	0.41	0.96	0.3(2.08)	1.09 to -0.49	4.38 to -3.77
bts_swing_phase	38(1.97)	38.3(2.55)	0.59	0.3 to 0.78	0.41	0.96	-0.3(2.08)	0.49 to -1.09	3.77 to -4.38
bts_single_support_phase	38.03(2)	38.32(2.54)	0.59	0.3 to 0.79	0.39	0.91	-0.29(2.07)	0.5 to -1.08	3.77 to -4.35
bts_double_support_phase	12.29(1.72)	11.84(2.04)	0.75	0.54 to 0.87	0.24	0.55	0.45(1.28)	0.94 to -0.03	2.96 to -2.06
bts_stride_length	1.37(0.11)	1.38(0.13)	0.83	0.68 to 0.92	0.04	0.09	-0.01(0.07)	0.02 to -0.04	0.13 to -0.15
bts_step_length	0.69(0.06)	0.69(0.06)	0.85	0.71 to 0.93	0.02	0.05	0(0.03)	0.01 to -0.02	0.06 to -0.07
bts_velocity	1.36(0.16)	1.38(0.18)	0.88	0.76 to 0.94	0.04	0.08	-0.02(0.08)	0.01 to -0.05	0.14 to -0.18
bts_cadence	118.57(8.89)	120.1(9.95)	0.90	0.8 to 0.95	0.27	0.63	-1.53(3.96)	-0.03 to -3.04	6.23 to -9.29

Table S4. Intra-rater measurement for BTS (Rater B)

Variables	1st measurement	2nd measurement	ICC		SEM	MDC(90%)	Bland-Altman Analysis		
	Mean (SD)	Mean (SD)	ICC	95% CI			Mean difference (SD)	95% CI	LOA superior - inferior
bts_stance_phase	61.32(2.06)	61.24(2.81)	0.62	0.34 to 0.8	0.45	1.04	0.08(2.17)	0.91 to -0.74	4.33 to -4.17
bts_swing_phase	38.65(2.1)	38.69(2.92)	0.62	0.34 to 0.8	0.47	1.09	-0.05(2.23)	0.8 to -0.9	4.33 to -4.42
bts_single_support_phase	38.7(2.3)	39.11(2.39)	0.83	0.67 to 0.92	0.10	0.24	-0.42(1.33)	0.09 to -0.92	2.18 to -3.01
bts_double_support_phase	11.79(2.45)	11.7(1.74)	0.56	0.26 to 0.77	0.47	1.10	0.09(2.02)	0.85 to -0.68	4.03 to -3.86
bts_stride_length	1.39(0.12)	1.4(0.16)	0.65	0.39 to 0.82	0.10	0.23	-0.01(0.12)	0.03 to -0.06	0.23 to -0.25
bts_step_length	0.69(0.06)	0.69(0.06)	0.86	0.72 to 0.93	0.02	0.05	0(0.03)	0.02 to -0.01	0.07 to -0.06
bts_velocity	1.38(0.18)	1.41(0.19)	0.83	0.67 to 0.91	0.02	0.05	-0.03(0.11)	0.01 to -0.07	0.18 to -0.24
bts_cadence	119.59(8.71)	121.49(10.1)	0.75	0.54 to 0.88	0.49	1.15	-1.9(6.49)	0.56 to -4.37	10.81 to -14.62

Table S5. Inter-rater measurements for Baiobit

Variables	Rater A	Rater B	ICC		SEM	MDC(90%)	Bland-Altman Analysis		
	Mean (SD)	Mean (SD)		95% CI			Mean difference (SD)	95% CI	LOA superior - inferior
bai_stance_phase	60.28(2.46)	60.7(1.57)	0.66	0.39 to 0.83	0.46	1.08	-0.42(1.69)	0.23 to -1.07	2.89 to -3.73
bai_swing_phase	39.72(2.47)	39.3(1.57)	0.66	0.39 to 0.83	0.47	1.09	0.41(1.69)	1.07 to -0.24	3.73 to -2.9
bai_single_support_phase	39.85(2.35)	39.73(1.72)	0.71	0.46 to 0.86	0.36	0.83	0.12(1.58)	0.73 to -0.5	3.22 to -2.98
bai_double_support_phase	20.44(4.77)	21.34(3.25)	0.70	0.46 to 0.85	0.57	1.32	-0.89(3.09)	0.3 to -2.09	5.16 to -6.95
bai_stride_length	1.51(0.2)	1.51(0.19)	0.83	0.67 to 0.92	0.04	0.09	0.01(0.12)	0.05 to -0.04	0.24 to -0.22
bai_step_length	0.76(0.1)	0.76(0.09)	0.82	0.65 to 0.91	0.03	0.07	0(0.06)	0.03 to -0.02	0.12 to -0.11
bai_velocity	1.32(0.2)	1.33(0.19)	0.87	0.74 to 0.94	0.02	0.04	-0.02(0.1)	0.02 to -0.05	0.18 to -0.21
bai_cadence	119.33(12.46)	120.7(10.84)	0.88	0.75 to 0.94	0.38	0.88	-1.37(5.78)	0.87 to -3.61	9.97 to -12.71

Table S6. Inter-rater measurements for BTS

Variables	Rater A	Rater B	ICC		SEM	MDC(90%)	Bland-Altman Analysis		
	Mean (SD)	Mean (SD)		95% CI			Mean difference (SD)	95% CI	LOA superior - inferior
bts_stance_phase	61.9(2.05)	61.34(2.23)	0.71	0.47 to 0.86	0.19	0.45	0.56(1.58)	1.17 to -0.05	3.65 to -2.52
bts_swing_phase	38.1(2.05)	38.64(2.24)	0.72	0.48 to 0.86	0.19	0.45	-0.55(1.56)	0.06 to -1.15	2.52 to -3.61
bts_single_support_phase	38.13(2.05)	38.8(2.21)	0.70	0.43 to 0.85	0.18	0.43	-0.68(1.58)	-0.06 to -1.29	2.42 to -3.77
bts_double_support_phase	12.12(1.78)	11.78(1.44)	0.55	0.23 to 0.76	0.33	0.77	0.34(1.54)	0.94 to -0.26	3.36 to -2.68
bts_stride_length	1.38(0.12)	1.39(0.13)	0.74	0.51 to 0.87	0.05	0.13	-0.01(0.09)	0.02 to -0.05	0.16 to -0.19
bts_step_length	0.69(0.06)	0.69(0.06)	0.80	0.62 to 0.9	0.02	0.04	0(0.04)	0.01 to -0.02	0.07 to -0.08
bts_velocity	1.37(0.17)	1.39(0.18)	0.83	0.67 to 0.92	0.04	0.09	-0.03(0.1)	0.01 to -0.07	0.17 to -0.22
bts_cadence	119.31(9.39)	120.81(8.89)	0.78	0.58 to 0.89	0.28	0.65	-1.5(6)	0.83 to -3.83	10.25 to -13.25
